# Verapamil Attenuates the Severity of Tendinopathy by Mitigating Mitochondrial Dysfunction through the Activation of the Nrf2/HO-1 Pathway

**DOI:** 10.3390/biomedicines12040904

**Published:** 2024-04-18

**Authors:** Zengguang Wang, Zhenglin Dong, Yiming Li, Xin Jiao, Yihao Liu, Hanwen Chang, Yaokai Gan

**Affiliations:** Department of Orthopedic Surgery, Shanghai Ninth People’s Hospital, Shanghai Jiao Tong University School of Medicine, No. 639 Zhi Zao Ju Road, Huangpu District, Shanghai 200011, China; wzg500189@163.com (Z.W.); forever-dzl@sjtu.edu.cn (Z.D.); jyliym0605@163.com (Y.L.); jiaoxin2020@126.com (X.J.); lyh19950227sjtu@sjtu.edu.cn (Y.L.); kfdoctorc@163.com (H.C.)

**Keywords:** verapamil, tendinopathy, ROS, Nrf2/HO-1 axis, mitochondrial dysfunction

## Abstract

Tendinopathy is a prevalent condition in orthopedics patients, exerting a profound impact on tendon functionality. However, its underlying mechanism remains elusive and the efficacy of pharmacological interventions continues to be suboptimal. Verapamil is a clinically used medicine with anti-inflammation and antioxidant functions. This investigation aimed to elucidate the impact of verapamil in tendinopathy and the underlying mechanisms through which verapamil ameliorates the severity of tendinopathy. In in vitro experiments, primary tenocytes were exposed to interleukin-1 beta (IL−1β) along with verapamil at a concentration of 5 μM. In addition, an in vivo rat tendinopathy model was induced through the localized injection of collagenase into the Achilles tendons of rats, and verapamil was injected into these tendons at a concentration of 5 μM. The in vitro findings highlighted the remarkable ability of verapamil to attenuate extracellular matrix degradation and apoptosis triggered by inflammation in tenocytes stimulated by IL−1β. Furthermore, verapamil was observed to significantly suppress the inflammation-related MAPK/NFκB pathway. Subsequent investigations revealed that verapamil exerts a remediating effect on mitochondrial dysfunction, which was achieved through activation of the Nrf2/HO-1 pathway. Nevertheless, the protective effect of verapamil was nullified with the utilization of the Nrf2 inhibitor ML385. In summary, the in vivo and in vitro results indicate that the administration of verapamil profoundly mitigates the severity of tendinopathy through suppression of inflammation and activation of the Nrf2/HO-1 pathway. These findings suggest that verapamil is a promising therapeutic agent for the treatment of tendinopathy, deserving further and expanded research.

## 1. Introduction

Tendinopathy, a condition within the realm of orthopedic pathologies, arises from the deleterious effects of excessive tendon usage. Its clinical manifestations primarily manifest as pain and compromised functionality [1]. Recent scientific investigations have unveiled a discernible occurrence of lower-limb tendinopathy within the general populace, estimating its prevalence to be within the modest range of 1 to 2% [2,3]. Despite persistent scholarly efforts, the intricate mechanisms of tendinopathy remain unclear. Many theories have been proposed in an attempt to elucidate the pathogenesis of tendinopathy, including the mechanical theory [4,5], apoptosis theory [6], inflammation theory [7], continuum model theory [8], and others. Our own research, along with the contributions of other esteemed investigators, has demonstrated that the inflammation and apoptosis of tenocytes, induced by oxidative stress, emerge as pivotal factors in the intricate tapestry of tendinopathy pathogenesis [9,10,11]. However, the mechanisms underlying oxidative stress generation and regulation in tendinopathy and the development of efficacious treatment modalities remain important subjects of investigation, necessitating further exploration and elucidation.

Mitochondria hold important significance as organelles within mammalian cells, as one of their main functions is the furnishing of ATP to sustain vital cellular activity via the process of oxidative phosphorylation [12]. Simultaneously, mitochondria serve as pivotal sites for the intracellular generation of reactive oxygen species (ROS) [13]. According to previous research, the majority of ROS (approximately 90%) can be traced back to the mitochondria [14]. When the functionality of mitochondria is impaired, the concentration of intracellular oxidative stress subsequently escalates [15].

In the presence of heightened oxidative stress, the intracellular antioxidant system is activated. Nuclear factor erythroid 2-related factor 2 (Nrf2), a transcription factor comprising seven conserved functional domains, named Neh1–Neh7 [16], is pivotal in orchestrating the adaptive cellular response to oxidative stress [17]. The activation of Nrf2 has been documented to mitigate oxidative stress by binding to antioxidant response elements (AREs) in the promoters of downstream antioxidant genes, such as heme oxygenase-1 (HO-1) [18]. As reported in the literature, Nrf2 knockout mice show an increased tendency to develop inflammation accompanied by extracellular matrix degradation [19]. Taken together, oxidative stress has emerged as a prominent player in the development of tendinopathy. To mitigate the inflammatory response and degradation of the tendon tissue, the modulation of Nrf2 expression holds promise as a potential therapeutic approach for the treatment of tendinopathy.

The present therapies for tendinopathy primarily focus on pain mitigation and functional enhancement, while little attention is given to addressing the underlying pathogenic mechanisms [10]. Verapamil is a commonly used calcium ion blocker in clinical practice, which is mostly used to treat cardiovascular diseases [20,21]. Recent scientific investigations have brought to light the distinctive therapeutic properties of verapamil, notably, its remarkable anti-inflammatory and antioxidant effects [22,23]. The scholarly work by Chen, Cao, and Pan et al. elucidated the fact that verapamil possesses the capacity to modulate anti-inflammatory and anti-oxidative pathways by regulating the translocation of Nrf2 to the nucleus [24]. Henceforth, verapamil emerges as a promising therapeutic agent for the management of tendinopathy. 

In this investigation, we postulated the potential therapeutic efficacy of verapamil in tendinopathy. Our in vitro experiments entailed an evaluation of the impact of verapamil on inflammation, apoptosis, and extracellular matrix remodeling in tenocytes. Furthermore, our in vivo experiments involved an assessment of histopathological alterations in tendons afflicted with tendinopathy in rat models. The objective of our endeavor was to interrogate the impact of verapamil in tendinopathy and explore the underlying mechanisms.

## 2. Materials and Methods

### 2.1. Study Design

Our investigation constituted a fundamental exploration of the efficacy of verapamil in the management of tendinopathy and the underlying mechanisms of its effects. This encompassed both in vivo and in vitro experiments. In the in vitro experiments, the tenocytes under study were categorized into three groups: the control group (addition of complete medium only), the IL−1β group (addition of complete medium containing 50 ng/mL IL−1β), and the IL−1β + verapamil group (addition of complete medium containing 50 ng/mL IL−1β and 5 μM verapamil). Similarly, for the in vivo experiments, the rats were stratified into four groups, denoted as the control group (injection of 50 μL of PBS only), the verapamil group (injection of 50 μL of PBS containing 5 μM verapamil), the tendinopathy group (injection of 50 μL of PBS containing 50 mg/mL collagenase type I), and the tendinopathy + verapamil group (injection of 50 μL of PBS containing 50 mg/mL collagenase type I and 5 μM verapamil). The sample size was determined in accordance with a previous report [25].

### 2.2. Animals

All animal experimentation carried out in this study received ethical approval from the Ethics Committee of Shanghai Ninth People’s Hospital, affiliated with the Shanghai Jiao Tong University School of Medicine (approval number SH9H-2023-A848-1). Sprague–Dawley rats, aged 8 weeks, were housed under appropriate conditions to induce tendinopathy. In accordance with previously established protocols [26], rats were subjected to an injection of 50 μL of 50 mg/mL collagenase type I (LS004196, Worthington Biochemical Corp., Lakewood, NJ, USA) into the tendon, followed by the concurrent administration of 50 μL of 5 μM verapamil three times a week. After two weeks, tendon tissues were meticulously collected. Subsequently, these tissue samples were subjected to various staining and immunohistochemistry techniques.

### 2.3. Materials and Reagents

Verapamil was obtained from Selleck Chemicals (S4202, Houston, TX, USA), and IL−1β was purchased from GenScript Technologies (Z03014, Piscataway, NJ, USA). Collagenase type I (LS004196, Worthington Biochemical Corp., Lakewood, NJ, USA) and ML385 (T4360, TargetMol, Shanghai, China) were also acquired. The antibodies employed included BAX (50599-2-Ig, Proteintech, Wuhan, China), BCL2 (26593-1-AP, Proteintech), MMP3 (17873-1-AP, Proteintech), MMP9 (10375-2-AP, Proteintech), MMP13 (18165-1-AP, Proteintech), COX2 (66351-1-Ig, Proteintech), β-actin (66009-1-Ig, Proteintech), IL6 (A11115, ABclonal, Wuhan, China), NRF2 (A21176, ABclonal), HO-1 (10701-1-AP, Proteintech), lamin B1 (12987-1-AP, Proteintech), P38 (14064-1-AP, Proteintech), P-P38 (28796-1-AP, Proteintech), ERK1/2 (11257-1-AP, Proteintech), P-ERK1/2 (28733-1-AP, Proteintech), P65 (10745-1-AP, Proteintech), P-P65 (AP0475, ABclonal), P-IκBα (AP0707, ABclonal), IκBα (A24909, ABclonal), anti-rabbit IgG (H+L) (DyLight™ 800 4X PEG conjugate) (5151, Cell Signaling Technology, Danvers, MA, USA), anti-mouse IgG (H+L) (DyLight™ 800 4X PEG conjugate) (5257, Cell Signaling Technology), anti-rabbit IgG (horseradish peroxidase (HRP) conjugate) (ab6721, Abcam, Cambridge, UK), anti-mouse IgG (horseradish peroxidase conjugate) (ab6789, Abcam), and anti-rabbit (Alexa Fluor^®^ 555 conjugate) (ab150078, Abcam).

### 2.4. Isolation and Culture of Tenocytes

In line with previous studies [11], Achilles tendons from Sprague–Dawley (SD) rats were surgically dissected and thoroughly cleansed via a 30 min immersion in 75% ethanol as a preparatory step. Subsequently, the Achilles tendons were immersed in Dulbecco’s modified Eagle’s medium (12320032, Gibco, Erie County, NY, USA), supplemented with 0.06% collagenase type I (LS004196, Worthington Biochemical Corp., Lakewood, NJ, USA), and incubated at a temperature of 37 °C for a duration of 12 h. Following this treatment, the solution was subjected to centrifugation for 5 min, after which the supernatant was carefully discarded. Then, the tendon tissue fragments were suspended and incubated in Dulbecco’s modified Eagle’s medium (12320032, Gibco), fortified with 10% fetal bovine serum (A5669701, Gibco) and 1% penicillin-streptomycin (C0222, Beyotime, Shanghai, China).

### 2.5. Cell Experiments 

The tenocytes were cultivated in culture plates and subsequently divided into three distinct groups: the control group, the IL−1β group, and the IL−1β + verapamil group. These groups were maintained at a temperature of 37 °C and a 5% CO_2_ atmosphere for a duration of 24 h. Following this incubation period, the control group was supplemented with fresh complete medium. The IL−1β group received IL−1β (50 ng/mL), while the IL−1β + verapamil group was treated with both IL−1β (50 ng/mL) and verapamil (5 μM) for an additional 24 h.

### 2.6. Cell Viability Assay

The cell viability of the tenocytes was assessed under varying concentrations of verapamil using the Cell Count Kit-8 (CCK-8) assay (C0037, Beyotime) following the guidelines provided by the manufacturer. The tenocytes were seeded in a 96-well plate at a density of 3000 cells per well and exposed to verapamil concentrations ranging from 0 to 5 μM for 24, 48, and 72 h. Subsequently, 100 μL of fresh complete medium containing 10% CCK8 reagent were added at specific time points, and the cells were subsequently incubated in a dark environment at a temperature of 37 °C for 1 h. The absorbance of the resulting solution at 450 nm was evaluated using an Infinite M200 Pro reader (Infinite M200 Pro, Tecan, Männedorf, Switzerland).

### 2.7. Live/Dead Cell Staining

To assess the toxicity of various concentrations of verapamil on tenocytes, a live/dead cell staining kit (KGA9501-1000, KeyGEN, Nanjing, China) was employed following the manufacturer’s instructions. A fluorescent microscope (DMi8, Leica Microsystems, Wetzlar, Germany) was utilized to observe the outcomes. Live cells exhibited a fluorescent green color, while dead cells displayed a distinct red color.

### 2.8. RNA Extraction and Quantitative Real-Time PCR Analysis

Tenocytes were cultivated in six-well plates at a density of 2 × 10^5^ cells per well for 24 h. Following the treatment, total RNA was extracted utilizing TRIzol reagent (Thermo Fisher Scientific, Waltham, MA, USA) in accordance with the provided instructions. Reverse transcription was carried out using PrimeScript RT Master Mix (RR036A, Takara, Shiga, Japan) according to the manufacturer’s instructions. The qPCR reaction was performed by employing SYBR Green (B21703, Bimake, Houston, TX, USA). The relative mRNA levels were determined using the 2^−ΔΔCt^ method with β-actin serving as the internal control. Please refer to Table 1 for the list of primers employed in this study.

### 2.9. RNA Sequencing and Differentially Expressed Gene Analysis

Tenocyte cells were cultured as described above. Total RNA was extracted from tenocytes using the TRIZOL reagent (Thermo Fisher Scientific) according to the manufacturer’s instructions. The Nanodrop ND-2000 system (Thermo Fisher Scientific) and Agilent Bioanalyzer 4150 system (Agilent Technologies, Santa Clara, CA, USA) were used to test the quality of samples. Then, mRNA purified from 1 μg of total RNA using Oligo dT-coated magnetic beads (19820ES50, Yeasen, Shanghai, China) was used to synthesize cDNA. The synthesized double-stranded cDNA fragments were then adapter-ligated for the preparation of the paired-end library. Adaptor-ligated cDNA was used for PCR amplification. PCR products were purified (AMPure XP system, Beckman Coulter, Brea, CA, USA) and the library quality was assessed on an Agilent Bioanalyzer 4150 system (Agilent Technologies). Finally, the library preparations were sequenced on DNBSEQ-T7 (MGI, Shenzhen, China). All of the data analysis was performed using an in-house pipeline (Shanghai Applied Protein Technology, Shanghai, China). Differential expression analysis was performed using the DESeq2 (http://bioconductor.org/packages/release/bioc/html/DESeq2.html) (accessed on 1 December 2023), and genes with |log2Fold change| > 1 and *p*-adjust < 0.05 were considered to be significantly differentially expressed genes. The clusterProfiler R software package (4.2.2) was used for Gene Ontology (GO) function enrichment and Kyoto Encyclopedia of Genes and Genomes (KEGG) pathway enrichment analysis. 

### 2.10. Protein Extraction and Western Blot Analysis

Tenocytes were seeded into a 6-well plate at a density of 2 × 10^5^ cells per well and subjected to the treatment described above. Subsequently, RIPA lysis buffer (P0013B, Beyotime) containing 1% protease–phosphatase inhibitor (P1045, Beyotime) was employed to lyse the cells. The resulting lysate was then centrifuged at 13,000× *g* for 15 min to separate the supernatant from the sediment. To prepare the protein samples for analysis, the supernatant was mixed with SDS-PAGE sample loading buffer (5×) (P0015, Beyotime) and boiled at 100 °C for 5 min. A nuclear protein extraction kit (P0027, Beyotime) was used to isolate nuclear proteins according to the manufacturer’s instructions. The proteins were then resolved by 4–20% SDS-PAGE and subsequently transferred onto 0.22 μm PVDF membranes (Merck-Millipore, Temecula, CA, USA). Following the transfer, the membranes were blocked with 5% non-fat milk (P0216, Beyotime) at room temperature for 2 h. Primary antibodies (BAX, 1:2000; BCL2, 1:1000; MMP3, 1:1000; MMP9, 1:1000; MMP13, 1:1000; β-actin, 1:20,000; IL6, 1:2000; NRF2, 1:500; HO-1, 1:1000; lamin B1, 1:5000; P38, 1:2000; P-P38, 1:1000; ERK1/2, 1:2000; P-ERK1/2, 1:1000; P65, 1:1000; P-P65, 1:500; P-IκBα, 1:500; IκBα, 1:2000) were then incubated with the membranes at 4 °C overnight. Subsequently, the membranes were incubated with secondary antibodies (anti-rabbit IgG (H+L) (DyLight™ 800 4X PEG conjugate), 1:5000; anti-mouse IgG (H+L) (DyLight™ 800 4X PEG conjugate), 1:5000) at room temperature for 1 h. The results were visualized using the Odyssey V3.0 image scanning system (Li-COR Inc, Lincoln, NE, USA).

### 2.11. Immunofluorescence

Tenocytes were cultured in a 24-well plate and subjected to the treatment described above. Following the treatment, the cells were washed three times with PBS and fixed with 4% paraformaldehyde (P0099, Beyotime) for 25 min. To enable permeabilization, PBS containing 0.2% Triton X-100 (P0096, Beyotime) was applied for 10 min, followed by three additional washes. Subsequently, a 5% BSA blocking solution (36102ES25, Yeasen) was applied at room temperature, with three PBS washes. Next, primary antibodies (MMP13, 1:50; HO-1, 1:50) were incubated with the blocked cells overnight. After three washes, fluorescent secondary antibodies (1:200) were incubated with the cells for 1 h at room temperature. Following a 15 min incubation with 4’,6-diamidino-2-phenylindole (DAPI) and subsequent three PBS washes, the results were visualized using a fluorescent microscope (DMi8, Leica Microsystems).

### 2.12. Reactive Oxygen Species Assay

The levels of ROS in the various tenocyte groups were assessed using the fluorescent probe dichlorodihydrofluorescein diacetate (DCFH-DA) (S0033S, Beyotime). Briefly, a diluted solution of DCFH-DA was prepared at a 1:1000 ratio in a serum-free culture medium, resulting in a final concentration of 10 µM. The cell culture medium was replaced with 1 mL of diluted DCFH-DA in each well of a six-well plate, ensuring comprehensive coverage of the cells. The cells were incubated for 20 min at 37 °C in a cell culture incubator. Subsequently, the cells were washed three times with serum-free culture medium and examined using a fluorescent microscope (Leica Microsystems).

### 2.13. Dihydroethidium Assay

The levels of superoxide anion in the various tenocyte groups were assessed using the fluorescent probe dihydroethidium (S0063, Beyotime). Dihydroethidium was used at a concentration of 2.5 μM, and the tenocytes were incubated at 37 °C for about 30 min, followed by three PBS washes, and examined using a fluorescent microscope (DMi8, Leica Microsystems).

### 2.14. Mitochondrial Membrane Potential Assay

A JC-1 kit (C2003S, Beyotime) was employed to assess the mitochondrial membrane potential. Tenocytes were seeded onto a Petri dish and subjected to the abovementioned treatment protocol. The JC-1 working staining solution was prepared in accordance with the manufacturer’s instructions. Subsequently, 1 mL of the JC-1 working staining solution was added to the Petri dish and incubated for 20 min at 37 °C. The cells were then washed twice with the JC-1 staining buffer. Finally, after a 15 min incubation with DAPI and three subsequent PBS washes, the fluorescence intensity was evaluated using a fluorescent microscope (DMi8, Leica Microsystems).

### 2.15. MitoTracker Green and MitoSOX Red Assay

MitoTracker Green (C1048, Beyotime) and MitoSOX (40778ES50, Yeasen) were employed for staining following the manufacturer’s specific instructions. Tenocytes were cultured in a Petri dish and subjected to the previously mentioned treatment. Initially, a 1 mM MitoTracker Green solution and a 5 mM MitoSOX solution were prepared using DMSO as the storage medium, and these working solutions were made at a ratio of 1:1000. Subsequently, the MitoTracker Green working solution was added to the Petri dish and incubated at 37 °C for 30 min. Then, the MitoSOX working solution was added to the Petri dish and incubated at 37 °C for 10 min. Following a 15 min incubation with DAPI and subsequent washing with PBS, the fluorescence intensity was assessed using a fluorescent microscope (DMi8, Leica Microsystems).

### 2.16. Histology Analysis

Tendon tissue samples from the animals were harvested and immersed in 4% paraformaldehyde for 48 h (*n* = 6 for independent biological replicates). Subsequently, the tendons were embedded in paraffin and subsequently sliced into 5 μm sections in Microtome (E0973, Beyotime). To evaluate the structural alterations in the tendon, the tissues were subjected to staining with hematoxylin and eosin (HE) as well as Masson’s trichrome staining. A histological grading system was employed to determine the histological score [27].

### 2.17. Immunohistochemistry

The sections were initially subjected to deparaffinization and rehydration. Endogenous peroxidases were blocked using 3% H_2_O_2_ for 30 min. After a 2 h block with 10% goat serum at room temperature, the sections were incubated with primary antibodies (MMP13, 1:50; COX2, 1:100; HO-1, 1:100; P-P38, 1:50; P-ERK1/2, 1:50; P-P65, 1:50) at 4 °C overnight. The following day, the sections were incubated with a secondary antibody conjugated with HRP (goat anti-rabbit IgG horseradish peroxidase conjugate, 1:500; goat anti-mouse IgG horseradish peroxidase conjugate, 1:500) for 2 h at room temperature after three washes in PBS. The images were obtained using a fluorescent microscope (DMi8, Leica Microsystems).

### 2.18. Terminal Deoxynucleotidyl Transferase-Mediated dUTP Nick-End Labeling

The paraffin sections were subjected to dewaxing in xylene for 5–10 min, followed by replacement with fresh xylene and an additional 5–10 min of dewaxing. Then, the paraffin sections were immersed in anhydrous ethanol for 5 min, 90% ethanol for 2 min, 70% ethanol for 2 min, and distilled water for 2 min. Following this, 20 μg/mL DNase-free proteinase K (ST532, Beyotime) were added and the sections were incubated at a temperature of 37 °C for 30 min. Subsequently, a terminal deoxynucleotidyl transferase-mediated dUTP nick-end labeling (TUNEL) stain (C1089, Beyotime) was added and the sections were incubated for 60 min in the absence of light. After a 15 min incubation with DAPI and subsequent washing with PBS, the fluorescence intensity was assessed using a fluorescent microscope (DMi8, Leica Microsystems).

### 2.19. Statistical Analysis

All data were derived from independent experiments (*n* = 3 or 6, respectively). The data are presented as the mean ± standard deviation (SD). The Brown–Forsythe test and Shapiro–Wilk test were used to test the homogeneity of variances and the normality of the distribution, respectively. Significance differences among the groups were determined using the one-way analysis of variance (ANOVA) with Tukey’s post hoc test as parametric tests. Significance differences among the groups were determined using the Kruskal–Wallis test with Dunn’s post hoc test as non-parametric tests. The analyses and figures were generated by employing Prism 8.0 (GraphPad Software Inc., San Diego, CA, USA) and SPSS22.0 (IBM, New York, NY, USA). A difference was deemed significant if the *p*-value was less than 0.05.

## 3. Results

### 3.1. Verapamil Has Little Toxicity in Tenocytes 

**Figure 1:**
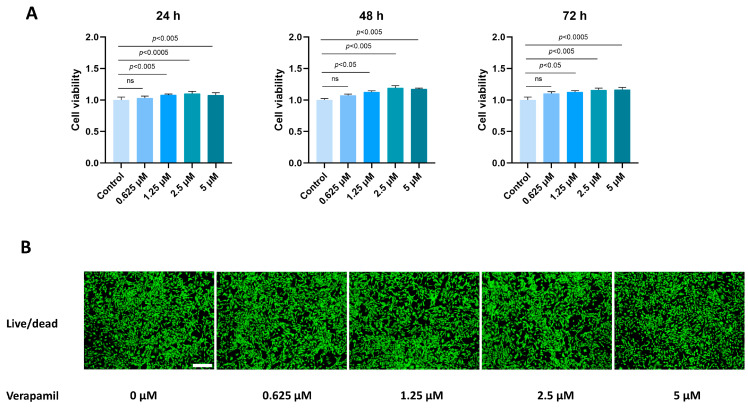
Toxicity of verapamil on tenocytes at different concentrations. (**A**) The effects of verapamil on cell viability were assessed via a CCK8 assay at 24, 48, and 72 h. (**B**) Live/dead cell staining of tenocytes treated with 0, 0.625, 1.25, 2.5, or 5 μM verapamil. Scale bar, 250 μm. Data are presented in the form of mean ± standard deviation, *n* = 3. A difference was deemed no significant (ns) if the *p*-value was greater than 0.05.

To assess the impact of verapamil on the viability of tenocytes, we employed the CCK8 assay. A total of 3000 cells were seeded into 96-well plates, and various concentrations of verapamil ranging from 0 to 5 μM were administered for 24, 48, and 72 h. The toxic effects of verapamil were then evaluated by comparing them to the control group. Notably, our results demonstrated that verapamil exhibited minimal toxicity toward tenocytes when used at a concentration of 0–5 μM (Figure 1A). Moreover, to further validate the lack of toxicity of verapamil within the 5 μM concentration range, we conducted live/dead cell staining after 24 h of exposure (Figure 1B). These analyses convincingly confirmed that verapamil exerted minimal toxic effects on tenocytes at concentrations between 0 and 5 μM. Based on these findings, we selected the concentration of 5 μM for subsequent experiments.

### 3.2. Verapamil Inhibits Extracellular Matrix Degradation, Inflammation, and Apoptosis

**Figure 2:**
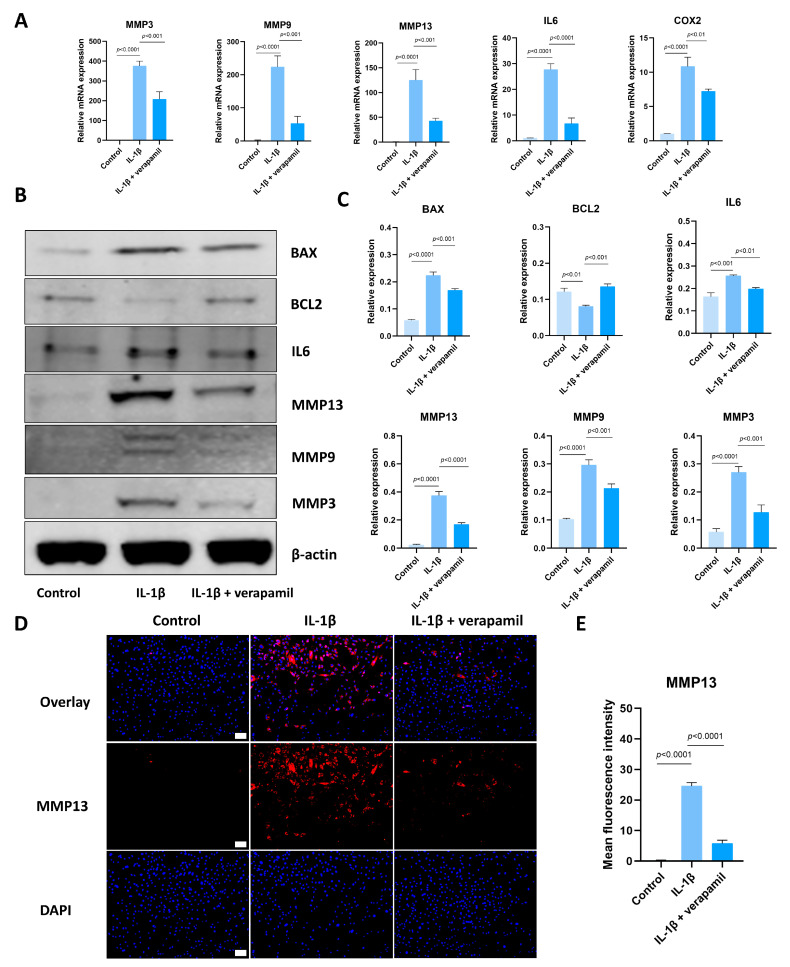
Verapamil protects tenocytes from extracellular matrix degradation, inflammation, and apoptosis induced by IL−1β. (**A**) The relative mRNA expression of IL6, COX2, MMP3, MMP9, and MMP13 analyzed via qPCR. (**B**) The protein level of BAX, BCL2, IL6, MMP3, MMP9, and MMP13 detected by means of Western blot. (**C**) Quantitative results of BAX, BCL2, IL6, MMP3, MMP9, and MMP13 detected by means of Western blot. (**D**) Immunofluorescent images of MMP13 obtained in combination with DAPI staining for the cell nucleus. Scale bar, 100 μm. (**E**) Quantitative results of MMP13 immunofluorescence. Data are presented in the form of mean ± standard deviation, *n* = 3.

Subsequently, we proceeded to investigate the impact of verapamil on the characteristic phenotype associated with tendinopathy, encompassing extracellular matrix degradation, inflammation, and apoptosis. Our qPCR analyses revealed that verapamil treatment effectively attenuated the upregulation of MMP3, MMP9, and MMP13 (Figure 2A), which are players closely associated with extracellular matrix degradation. Moreover, the administration of verapamil led to significant inhibition of the expression of inflammatory factors, namely, IL6 and COX2 (Figure 2A). These promising findings were further corroborated by Western blot, which demonstrated a substantial suppression of the protein levels of MMP3, MMP9, MMP13, and IL6 (Figure 2B,C). Moreover, immunofluorescent staining of MMP13 substantiated our qPCR and Western blot results (Figure 2D,E). Lastly, we sought to evaluate the impact of verapamil on markers of apoptosis. Through Western blot, we observed a rescue of BCL2 and the inhibition of BAX expression upon verapamil treatment (Figure 2B,C). These results suggest that verapamil rescues IL−1β-induced inflammation, apoptosis, and extracellular matrix degradation in tenocytes.

### 3.3. The Transcriptomic Evaluation of Tendinopathy

**Figure 3:**
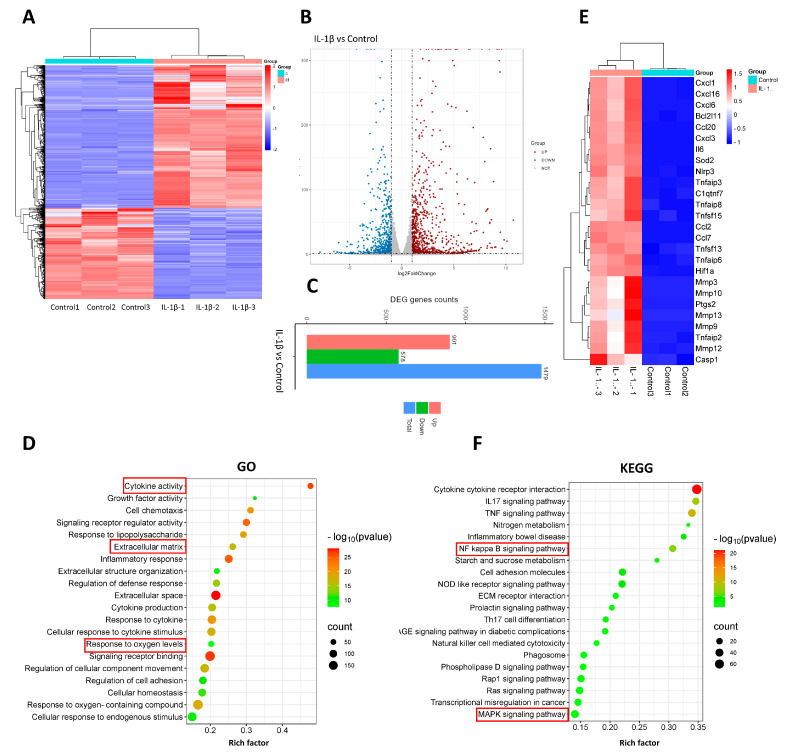
RNA sequencing and analysis comparing normal tenocytes with those stimulated by IL −1β. (**A**) Heatmap of genes differentially expressed between normal tenocytes and those stimulated with IL−1β. (**B**) Volcanol plot of genes differentially expressed between normal tenocytes and those stimulated by IL−1β. (**C**) Chart of differentially upregulated and downregulated genes between normal tenocytes and those stimulated with IL−1β; genes with |log2FC| > 1 and *p*-adjust < 0.05 were considered to be significantly different expressed genes. (**D**) Gene Ontology (GO) enrichment analysis of differentially expressed genes; rich factor is the ratio of differentially expressed protein number annotated in this pathway term to all protein number annotated. (**E**) Inflammatory activity-, apoptosis-, extracellular matrix degradation-, and oxidative stress-related genes. (**F**) Kyoto Encyclopedia of Genes and Genomes (KEGG) pathway analysis; rich factor is the ratio of differentially expressed protein numbers annotated in this pathway term to all annotated protein numbers. *n* = 3.

In order to elucidate the primary alterations associated with tendinopathy in comparison to the normal group, we conducted RNA sequencing analysis to identify differentially expressed genes. These results were visualized through the utilization of heatmaps and volcano plots (Figure 3A,B). Remarkably, the analysis revealed a total of 1479 genes that exhibited significant differential expression, comprising 901 upregulated and 578 downregulated genes (Figure 3C). To further comprehend the functional consequences of these differentially expressed genes in tenocytes, we performed GO and KEGG pathway analyses. The outcomes of the GO analysis demonstrated a notable enrichment of processes related to inflammation and extracellular matrix regulation (Figure 3D). Subsequent investigation revealed a substantial increase in the expression of genes associated with inflammation, including IL6, COX2, and similar genes, as well as of genes implicated in extracellular matrix degradation, such as MMP3, MMP9, and MMP13 (Figure 3E). These findings signify the successful induction of our experimental model. Subsequent research showed that the homeostasis of oxidative stress was disrupted, with pivotal elements in the pro-oxidant and antioxidant stress response, such as HIF1α and SOD2, experiencing a marked elevation (Figure 3E). In addition, the KEGG pathway analysis revealed a notably distinctive engagement of the NFκB/MAPK pathway (Figure 3F). 

### 3.4. The Effect of Verapamil on the NFκb and MAPK Pathways 

**Figure 4:**
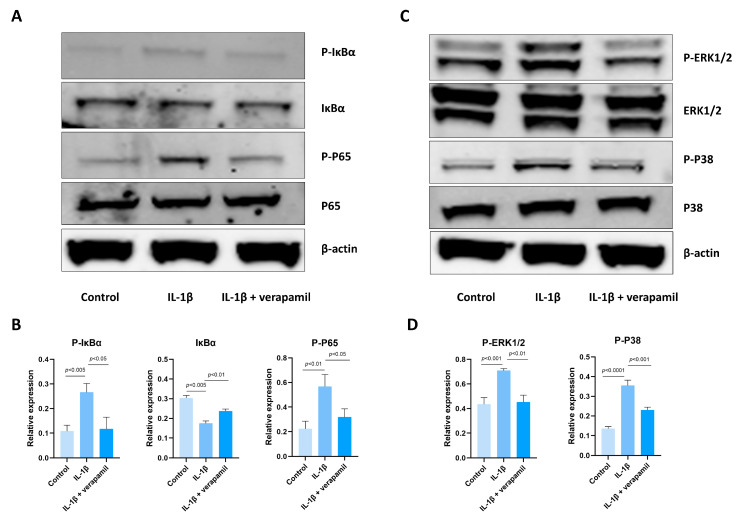
Expression of the NFκB and MAPK signaling pathways after IL−1β and verapamil administration. (**A**) The phosphorylation levels of IκBα and P65 in tenocytes of the control, IL−1β, and IL−1β + verapamil groups examined by means of Western blot. (**B**) Quantitative results of phosphorylation levels of P65 and IκBα and quantitative results of IκBα. (**C**) The phosphorylation levels of P38 and ERK1/2 in tenocytes of the control, IL−1β, and IL−1β + verapamil groups examined by means of Western blot. (**D**) Quantitative results of phosphorylation levels of P38 and ERK1/2. Data are presented in the form of mean ± standard deviation, *n* = 3.

The involvement of the NFκB and MAPK pathways in inflammation and apoptosis has been well documented in the literature [28,29], and our sequencing results showed that the NFκB and MAPK pathways are enriched in tenocytes stimulated by IL−1β. Hence, we conducted an analysis to assess the alterations in the expression levels of proteins associated with these pathways. Remarkably, the outcomes obtained from Western blots indicated a substantial upregulation of the core proteins P-P65, P-IκBα (Figure 4A,B), and P-ERK1/2, P-P38 (Figure 4C,D), which serve as vital indicators of the functional NFκB and MAPK pathways, respectively. Importantly, our subsequent investigations revealed that the administration of verapamil effectively attenuated this observed trend. Collectively, these results suggest that verapamil inhibits the activation of MAPK and NFκB pathways in IL−1β-induced tenocytes.

### 3.5. Verapamil Rescues Mitochondrial Dysfunction and Reactive Oxygen Species Production in Tendinopathy

**Figure 5:**
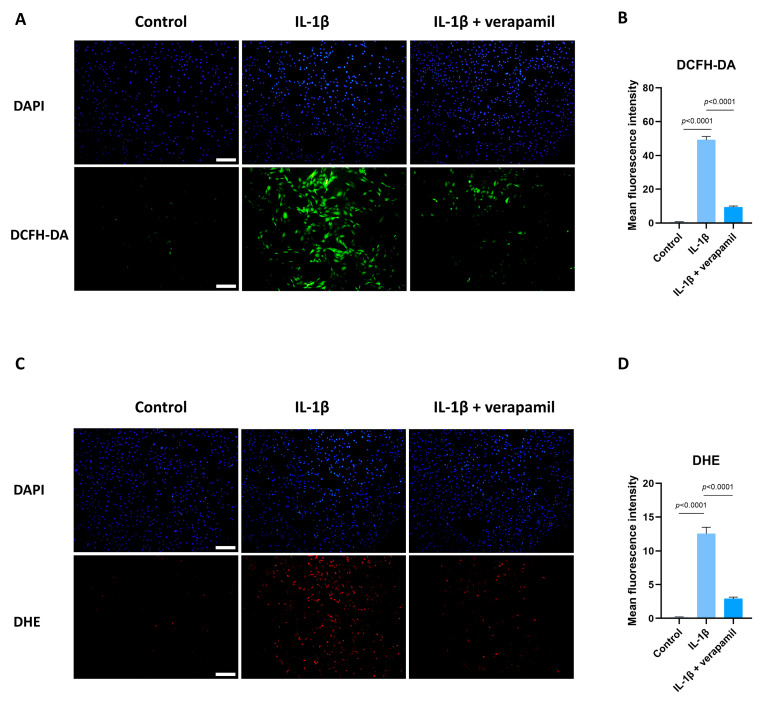
Protective effect of verapamil on IL−1β-induced oxidative stress in tenocytes. (**A**) DCFH-DA probe and DAPI staining for nucleus. Scale bar, 200 μm. (**B**) Quantitative results of DCFH-DA. (**C**) Dihydroethidium probe and DAPI staining for nucleus. Scale bar, 200 μm. (**D**) Quantitative results of DHE. Data are presented in the form of mean ± standard deviation, *n* = 3.

**Figure 6:**
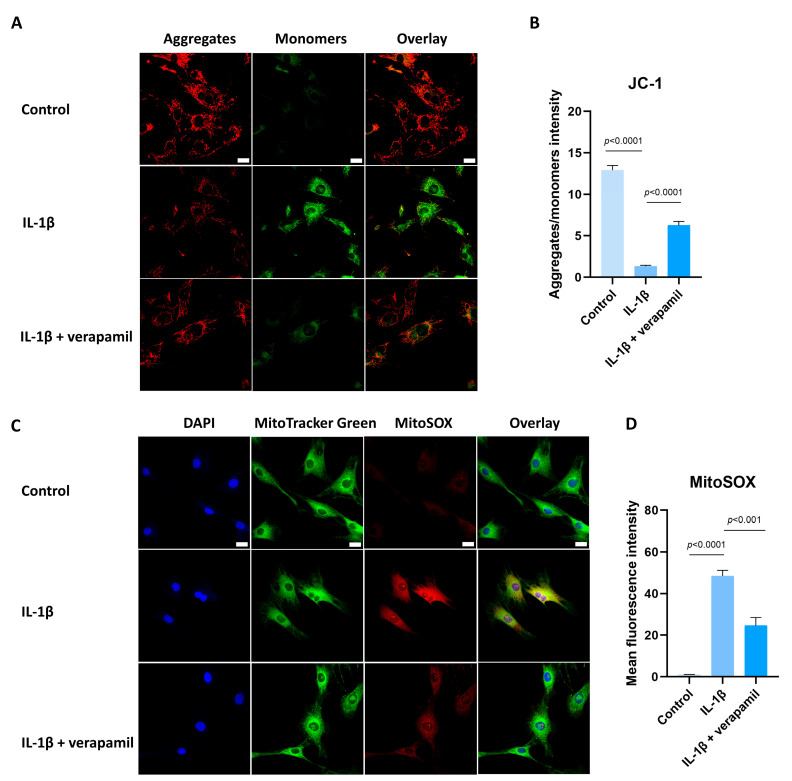
Protective effect of verapamil on IL−1β-induced mitochondrial dysfunction in tenocytes. (**A**) Fluorescent images of JC-1 staining. Scale bar, 20 μm. (**B**) Quantitative results of JC-1. (**C**) MitoTracker Green staining for the mitochondria and MitoSOX probe for the superoxide combined with DAPI staining for the nucleus. Scale bar, 20 μm. (**D**) Quantitative results of MitoSOX. Data are presented in the form of mean ± standard deviation, *n* = 3.

Previous reports suggested that oxidative stress is closely related to extracellular matrix degradation, inflammation, and apoptosis [24,30,31]. Consequently, ROS levels were assessed by employing the DCFH-DA fluorescent probe. The findings revealed a substantial increase in ROS prompted by IL−1β, while verapamil was able to reverse this increase (Figure 5A,B). In accordance with these outcomes, the superoxide anion probe dihydroethidium (DHE) exhibited a parallel pattern (Figure 5C,D). Collectively, these findings strongly point to the antioxidant efficacy of verapamil. It is noteworthy that mitochondria serve as pivotal sites for intracellular ROS generation. Detecting changes in the mitochondrial membrane potential is crucial for evaluating mitochondrial function. In this study, the JC-1 probe was employed to effectively ascertain alterations in the mitochondrial membrane potential. The results unequivocally demonstrated a sharp decrease in mitochondrial membrane potential (Figure 6A,B), which was mitigated by verapamil intervention. Furthermore, verapamil treatment led to a notable inhibition of mitochondrial superoxide production compared to IL−1β stimulation (Figure 6C,D). Taken together, these fluorescent microscopy data underscore the potential of verapamil to suppress IL−1β-induced oxidative stress by enhancing mitochondrial function.

### 3.6. The Nrf2/HO-1 Pathway Plays an Important Role in Verapamil Protective Effects

**Figure 7:**
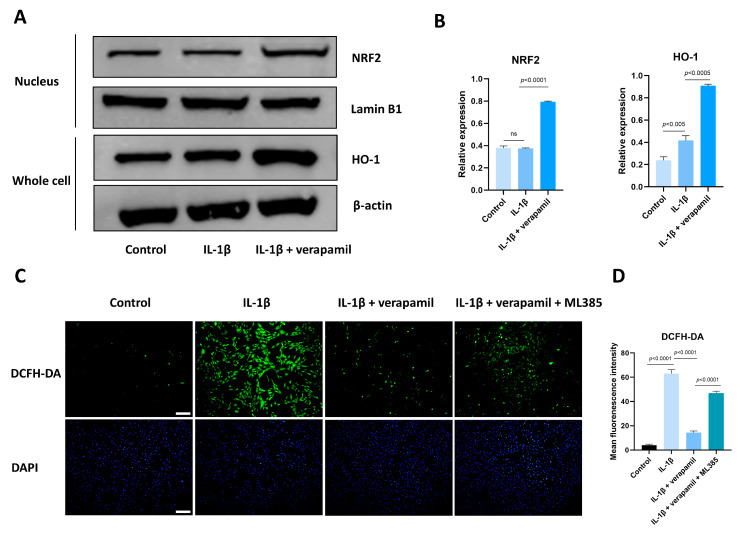
Verapamil facilitates Nrf2 entry into the nucleus to exert its protective effects. (**A**) The levels of Nrf2 translocation to the nucleus and the expression level of HO-1 in tenocytes of the control, IL−1β, and IL−1β + verapamil groups examined by Western blot. (**B**) Quantitative results of Nrf2 and HO-1. (**C**) DCFH-DA probe and DAPI staining for nucleus among the control, IL−1β, IL−1β + verapamil, and IL−1β + verapamil + 2 μM ML385 groups. Scale bar, 200 μm. (**D**) Quantitative results of DCFH-DA. Data are presented in the form of mean ± standard deviation, *n* = 3.

**Figure 8:**
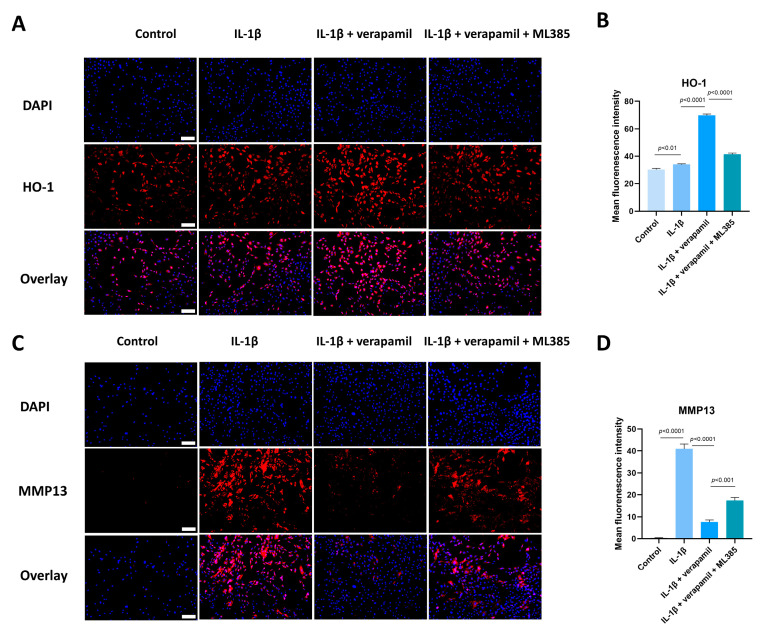
ML385 reverses the protective effect of verapamil. (**A**) Immunofluorescent images of HO-1 obtained in combination with DAPI staining for the cell nucleus among the control, IL−1β, IL−1β + verapamil, and IL−1β + verapamil + 2 μM ML385 groups. Scale bar, 150 μm. (**B**) Quantitative results of HO-1 staining among the control, IL−1β, IL−1β + verapamil, and IL−1β + verapamil + 2 μM ML385 groups. (**C**) Immunofluorescent images of MMP13 obtained in combination with DAPI staining for the cell nucleus among the control, IL−1β, IL−1β + verapamil, and IL−1β + verapamil + 2 μM ML385 groups. Scale bar, 150 μm. (**D**) Quantitative results of MMP13 staining among the control, IL−1β, IL−1β + verapamil, and IL−1β + verapamil + 2 μM ML385 groups. Data are presented in the form of mean ± standard deviation, *n* = 3.

**Figure 9:**
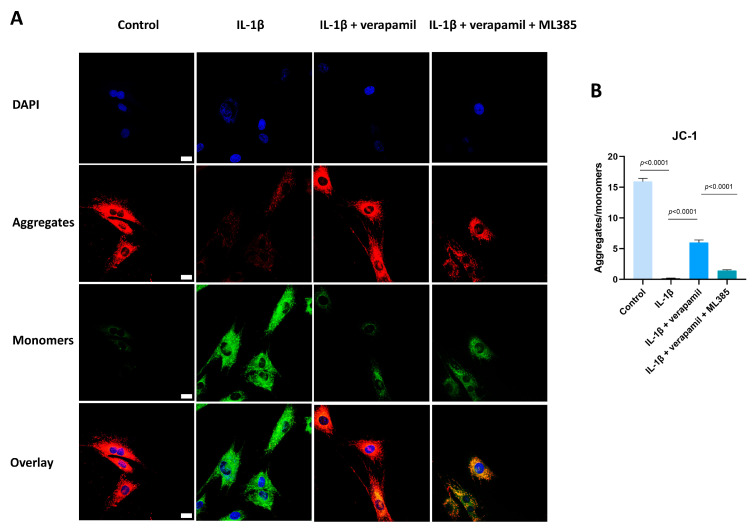
ML385 has the capacity to reverse the protective function of verapamil on the mitochondrial membrane potential. (**A**) Fluorescent images of JC-1 staining among the control, IL−1β, IL−1β + verapamil, and IL−1β + verapamil + 2 μM ML385 groups. (**B**) Quantitative results of JC-1 staining among the control, IL−1β, IL−1β + verapamil, and IL−1β + verapamil + 2 μM ML385 groups. Scale bar, 20μm. Data are presented in the form of mean ± standard deviation, *n* = 3.

Given that the Nrf2/HO-1 intracellular pathway is an important safeguard against oxidative stress, we analyzed the expression of Nrf2 and HO-1. Western blot suggested that the expression levels of Nrf2 in the nucleus displayed no significant change, but its downstream target gene HO-1 was upregulated slightly when stimulated by IL−1β, which meant that the intracellular antioxidant response system was activated. When treated with verapamil, the nuclear levels of Nrf2 and HO-1 were upregulated significantly (Figure 7A,B). To ascertain whether verapamil executes its protective influence through Nrf2, we employed ML385, an Nrf2 inhibitor, to repress the conventional functionality of Nrf2. Our findings disclosed an enhancement in ROS levels following ML385 treatment compared to the verapamil-only treatment group (Figure 7C,D). Subsequent investigations demonstrated that ML385 application inhibited the expression of HO-1 and elevated the level of MMP13 compared to the IL−1β + verapamil group (Figure 8) and precipitated a significant decline in mitochondrial membrane potential (Figure 9). Collectively, this body of evidence highlights the protective role that verapamil plays by facilitating the translocation of Nrf2 into the nucleus and activating the Nrf2/HO-1 pathway.

### 3.7. Verapamil Rescues the Severity of Achilles Tendinopathy In Vivo

**Figure 10:**
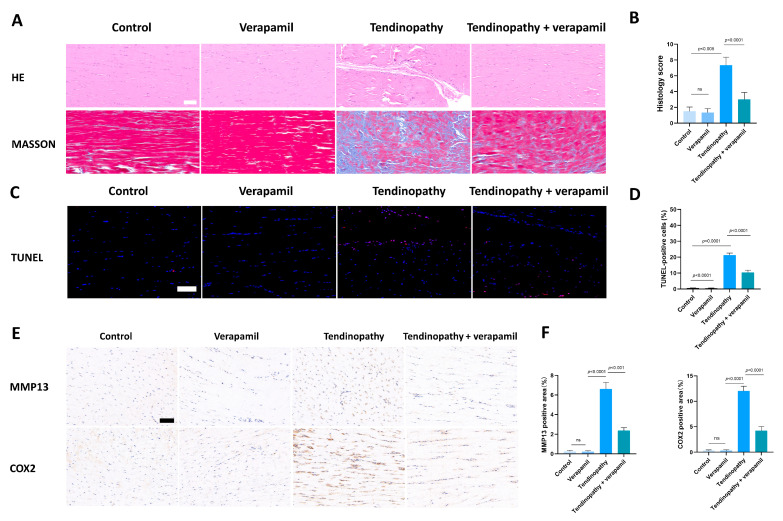
Evaluation of the protective effect of verapamil on rat tendons in vivo. (**A**) HE and Masson’s trichrome staining of tendons. Scale bar, 100 μm. (**B**) Histology score of HE staining among the four groups. Scale bar, 100 μm. (**C**) TUNEL staining among the four groups. (**D**) Quantitative results of TUNEL staining. (**E**) Immunohistochemical staining of MMP13 and COX2 among the four groups. Scale bar, 100 μm. (**F**) Quantitative results of immunohistochemical staining of MMP13 and COX2. Data are presented in the form of mean ± standard deviation, *n* = 6.

**Figure 11:**
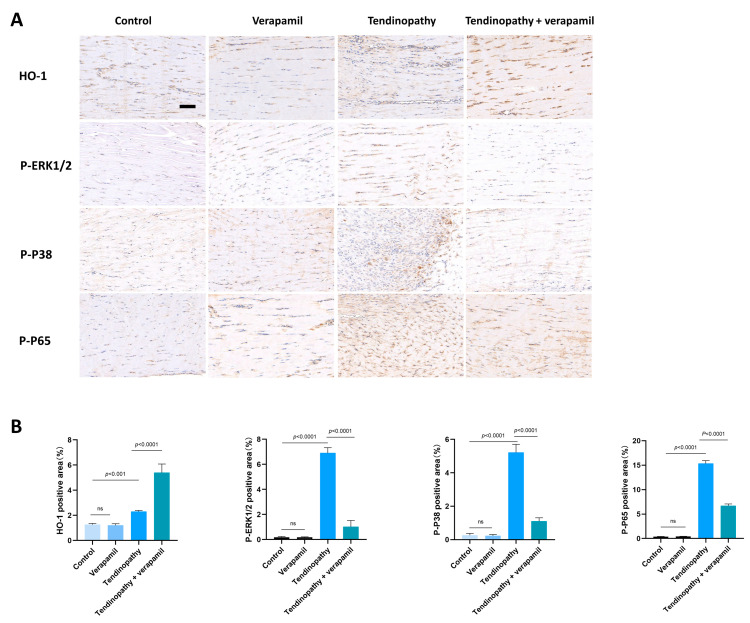
Evaluation of the protective effect of verapamil on rat tendons in vivo. (**A**) Immunohistochemical staining of HO-1, P-ERK1/2, P-P38, and P-P65 among the four groups. Scale bar, 100 μm. (**B**) Quantitative results of immunohistochemical staining of HO-1, P-ERK1/2, P-P38, and P-P65. Data are presented in the form of mean ± standard deviation, *n* = 6.

Finally, we conducted an assessment of the protective effects of verapamil administration in a collagenase-I-induced tendinopathy model. Histological analysis using HE and Masson’s trichrome staining revealed a pronounced disorganization and lax arrangement of collagen fibers in the tendinopathic tendon compared to the control group. Remarkably, verapamil treatment successfully ameliorated the structural disorder of collagen fibers in the tendinopathic tendon, and the whole histological damage decreased on the whole (Figure 10A,B). Additionally, subsequent TUNEL staining studies revealed a heightened proportion of apoptotic cells in tendinopathy tissues, which was mitigated by the injection of verapamil (Figure 10C,D). Furthermore, we employed immunohistochemical staining to investigate the expression levels of inflammatory, extracellular matrix degradation, and antioxidant markers. Our findings indicated a significant increase in the expression of matrix metalloproteinase 13 (MMP13) and inflammatory factors, such as cyclooxygenase 2 (COX2), in the collagenase-I-induced group as compared to the control group, and this trend was reversed by verapamil treatment (Figure 10E,F). Consistent with these results, activation of the NFκB and MAPK pathways was inhibited when treated with verapamil compared to the tendinopathy group (Figure 11A,B). In addition, the antioxidant gene (HO-1) was upregulated during tendinopathy, which means that the body’s autonomous antioxidant system was activated, and verapamil treatment significantly increased the level of HO-1 (Figure 11A,B). Taken together, these results provide compelling evidence that verapamil administration exerts a rescuing effect on the injured Achilles tendon in our experimental model of tendinopathy.

## 4. Discussion

In our investigation, verapamil, which at a concentration of 5 μM showed great biocompatibility, exhibited the ability to attenuate the levels of inflammation (IL6 and COX2), extracellular matrix degradation (MMP3, MMP9, and MMP13), and apoptosis (BCL2 and BAX). Moreover, we reported the protective effects of verapamil on mitochondria, which, when damaged, cause high levels of oxidative stress. Further results showed that the Nrf2/HO-1 pathway was activated by verapamil, mediating its protective potential against oxidative stress. Therefore, our results indicate that verapamil is a valuable drug for the therapy of tendinopathy.

Tendinopathy, an exercise-associated degenerative disorder, is characterized by heightened levels of inflammation and the degradation of collagen [26]. The etiology of tendinopathy remains elusive, despite a multitude of theories being posited, including mechanical, inflammation, vascular, and neurogenic hypotheses, as well as the continuum model [4,32,33,34,35]. An increasing body of research underscores the pivotal role that inflammation plays in the progression of tendinopathy [36]. However, mechanisms of inflammation in tendinopathy remain undefined and the effectiveness of current medications for tendinopathy remains questionable [37]. Consequently, it has become imperative to elucidate the underlying mechanisms of inflammation in tendinopathy and identify targeted therapeutic strategies. 

Verapamil, a pharmaceutical agent frequently employed in the management of cardiovascular ailments, has recently garnered attention. Plenty of contemporary studies have highlighted its distinct role in attenuating inflammation and providing antioxidant benefits in a variety of pathologies, including intervertebral disc degeneration, hepatic inflammation, acute liver failure, and hyperglycemic stroke, among others [24,38,39,40], but little is known about its efficacy in tendinopathy.

As integral constituents of the tendon, the perturbed molecular biology of tenocytes in pathological conditions mirrors the holistic alterations observed in tendinopathy. Consequently, we undertook RNA sequencing in tenocytes stimulated by IL−1β versus the control group. The GO results illustrated an enrichment of inflammation and extracellular matrix-related processes. Moreover, the KEGG pathway analysis revealed a marked enrichment of the NFκB and MAPK pathways. Further analysis indicated a significant upregulation of genes associated with inflammation, extracellular matrix degradation, and oxidative stress. All of these findings are in synchrony with previous studies, corroborating the successful establishment of our tendinopathy cell model and indicating potential areas for intervention in subsequent research [10,41].

Employing RNA sequencing, we deciphered the involvement of multiple pathways in orchestrating inflammatory responses, the particular highlight of which were the signaling pathways of NFκB and MAPK, which are considered to be strongly linked to inflammatory response [29]. The MAPK family, with astonishing precision, orchestrates the conveyance of signals through a complex phosphorylation cascade, thereby meticulously regulating an array of cellular biological processes [42]. NF-κB is not an isolated gene but rather a closely related family of transcription factors, comprising five distinct genes, including NF-κB1 (p50/p105), NF-κB2 (p52/p100), RelA (p65), c-Rel, and RelB. These genes engender seven proteins, and within the canonical NFκB pathway, a dimer is formed by p50 and p65, remaining inert due to the presence of IκBα. Upon initiation by inflammatory stimuli, IκBα undergoes phosphorylation, followed by degradation via the proteasome. This sequence of events facilitates the translocation of the dimer to the nucleus, where it executes its function [43]. The activation of the NFκB and MAPK pathways can upregulate the expression of the MMP family and inflammatory cytokines [44,45]. Our study shows that NFκB and MAPK are associated with the development of rat tendinopathy and that verapamil possesses the capacity to impede the activation of the NFκB/MAPK signaling pathways. This discovery underscores a distinct pathway through which verapamil ameliorates the phenotype associated with tendinopathy.

An increasing number of studies have demonstrated that oxidative stress is involved in the development of tendinopathy [46,47,48]. A recent clinical study showed that superoxide-induced oxidative stress may be an important risk factor for retearing after arthroscopic rotator cuff repair (ARCR) [49]. Oxidative stress is strongly associated with the progression of inflammation, apoptosis, and extracellular matrix degradation [30,50,51,52]. Mitochondrial dysfunction contributes to the production of ROS. Mitochondrial reactive oxygen species (mtROS) predominantly constitute the principal source of free radicals within the majority of cell types [53]. Our results indicate that the intracellular and mitochondrial ROS levels were elevated and the mitochondrial membrane potential was decreased in tenocytes stimulated by IL−1β. Verapamil administration can reverse the decrease in mitochondrial membrane potential and inhibit ROS production. In conjunction with the investigation of ROS production, we delved into the realm of intracellular ROS eradication mechanisms. 

The nuclear factor erythroid 2-related factor 2 (Nrf2) functions as a critical regulator of intracellular antioxidant mechanisms and forms an association with the antioxidant response element (ARE), prompting the upregulation of antioxidant genes that include NAD(P)H quinone oxidoreductase 1 (NQO1) and (HO-1) [54]. Nrf2 is activated mainly by entering the cell nucleus, and the translocation of Nrf2 is regulated by two main pathways, the canonical and non-canonical mechanisms [55]. In the canonical pathway, the Keap1–Nedd8–Cul3–Rbx1 complex sequesters the Nrf2 protein and facilitates the transfer of ubiquitin (Ub) proteins to the Neh2 domain in Nrf2. Subsequently, ubiquitinated Nrf2 is transferred to the 26S proteasome for degradation. In the non-canonical pathway, some proteins, such as P62 [56], DPP3 [57], BRCA1 [58], and others, possess the capacity to disrupt the Keap1–Nrf2 complex through direct interaction with Keap1 or Nrf2. This disruption inhibits Nrf2 ubiquitination and its subsequent 26S proteasomal degradation, leading to its nuclear translocation [59]. 

HO-1 represents an antioxidant microsomal enzyme that is a member of the heat shock protein family [60]. The upregulation of HO-1 expression by Nrf2 assumes a crucial role within the cellular antioxidant defense mechanism through the degradation of hemoglobin, myoglobin, and cytochrome *c* [61,62]. The Nrf2/HO-1 signaling cascade stands out as a pivotal intracellular antioxidant pathway, the activation of which serves to shield cells against the detrimental effects of heightened oxidative stress and the ensuing inflammatory response [63]. Researchers have demonstrated that activation of the Nrf2/HO-1 pathway assumes a critical role in the management of numerous diseases, including Parkinson’s disease, intracerebral hemorrhage (ICH), osteoarthritis, and others [64,65,66]. 

We conducted an analysis on the state of Nrf2 within the nucleus, during which our observations made it clear that the extent of nuclear translocation experienced no significant augmentation when stimulated with IL−1β, but the downstream genes, such as HO-1, increased slightly. This patterning suggests a disruption in the equilibrium of intracellular oxidative stress, implying spontaneous intracellular regulation to maintain homeostasis. The addition of verapamil emphatically amplified the translocation magnitude of Nrf2 to the nucleus and the expression of its corresponding downstream gene HO-1. Furthermore, the application of Nrf2 inhibitor ML385 reversed the protective effect of verapamil, leading to increased levels of intracellular oxidative stress and inflammation, as well as impaired mitochondrial function. This compelling evidence underscores the fact that verapamil exerts its antioxidant effects by activating the Nrf2/HO-1 pathway. 

Our in vivo studies reinforced these revelations, illustrating that the administration of verapamil ameliorates the histological manifestations of tendinopathy. Further, a significant elevation in in vivo antioxidant levels was observed when treating with verapamil, signaling a marked improvement in the cellular redox balance. It is important to note that when using verapamil alone in vivo, the expression of HO-1 displays no significant change, indicating that verapamil has little antioxidant impact on healthy tendons. This, in tandem with the suppression of inflammation and extracellular matrix degradation, underscores the potential therapeutic efficacy of verapamil in managing tendinopathy.

Verapamil, a phenylalkylamine derivative, acts as an antagonist of Ca^2+^ influx through the slow channels of vascular smooth muscle and cardiac cell membranes, thereby exerting a pronounced hypotensive effect [67]. Recent investigations have expanded the potential therapeutic applications of verapamil to other medical conditions. A recent clinical trial showed that verapamil is a safe and effective novel intervention in type 1 diabetes [68]. Consistently, our study demonstrates the efficacy of verapamil in inhibiting the onset and progression of tendinopathy in rats, presenting a novel avenue for the clinical utilization of this compound. Both localized injections and external dressings emerge as promising modes of verapamil administration, warranting further exploration in upcoming clinical trials. 

Our study possesses certain limitations that deserve acknowledgement. For instance, we have yet to fully decipher the intricate mechanism underpinning the mitochondrial dysfunction in tenocytes triggered by IL−1β. This aspect remains a fertile ground for further exploration and warrants comprehensive scrutiny.

## 5. Conclusions

Our results demonstrate the influence of verapamil in the remediation of tendinopathy. Our in vitro experiments revealed that verapamil effectively prevents inflammation-mediated extracellular matrix degradation, and apoptosis of tenocytes while simultaneously suppressing the MAPK and NFκB pathways. Mechanistically, verapamil demonstrated a protective role in mitigating mitochondrial damage by activating the Nrf2/HO-1 pathways. Furthermore, our in vivo experiments confirmed that verapamil restores the histological phenotype of Achilles tendinopathy in rats. Consequently, these findings indicate the substantial clinical potential of verapamil, which merits further study.

## Figures and Tables

**Table 1:** Primer base sequences for qPCR detection.

Gene	Forward Primer (5′–3′)	Reverse Primer (5′–3′)
*β* *-* *actin*	GTC CAC CCG CGA GTA CAA C	GGA TGC CTC TCT TGC TCT GG
*MMP3*	GCT GTC TTT GAA GCA TTT GGG TT	CCT CCA TGA AAA GAC TCA GAG GA
*MMP9*	TCC AGC ATC TGT ATG GTC GTG	GCA GTG GGA CAC ATA GTG GG
*MMP13*	CAA GCA GCT CCA AAG GCT AC	TGG CTT TTG CCA GTG TAG GT
*IL6*	CCA GTT GCC TTC TTG GGA CT	TGC CAT TGC ACA ACT CTT TTC
*COX2*	CTC AGC CAT GCA GCA AAT CC	GGG TGG GCT TCA GCA GTA AT

## Data Availability

The raw data supporting the conclusions of this article will be made available by the authors on request. The RNA sequencing data can be found in the GEO database (GSE253913).
